# List of Authors

**DOI:** 10.2188/jea.JE20220295

**Published:** 2022-12-05

Authors’ affiliations and their roles in the supervisory activities of the Fukushima Health Management Survey as of fiscal year 2022 (in square brackets)

^*^ Affiliation 1 denotes the main affiliation

**Table:** 

(Alphabetical order)
**Akahane, Keiichi**
1. National Institutes for Quantum Science and Technology
[A member of the Basic Survey and Radiation Dose Estimates Expert Committee]

**Fujimori, Keiya**
1. Department of Obstetrics and Gynecology, Fukushima Medical University School of Medicine
2. Office of the Pregnancy and Birth Survey, Radiation Medical Science Center for the Fukushima Health Management Survey, Fukushima Medical University
[A member of the Pregnancy and Birth Survey Expert Committee, the Risk Communication Evaluation Expert Committee, and the Database System Expert Committee]

**Goto, Aya**
1. Center for Integrated Science and Humanities, Fukushima Medical University
2. Office of the Pregnancy and Birth Survey, Radiation Medical Science Center for the Fukushima Health Management Survey, Fukushima Medical University
[A member of the Pregnancy and Birth Survey Expert Committee]

**Goto, Saori**
1. Seiwa Junior College
2. Radiation Medical Science Center for the Fukushima Health Management Survey, Fukushima Medical University
[An observer of the Mental Health/Life Style Survey and Care Expert Committee]

**Harigane, Mayumi**
1. Office of the Mental Health and Lifestyle Survey and Care, Radiation Medical Science Center for the Fukushima Health Management Survey, Fukushima Medical University
2. Department of Public Health, Fukushima Medical University School of Medicine
[A member of the Mental Health/Life Style Survey and Care Expert Committee]

**Hashimoto, Shigeatsu**
1. Department of Endocrinology, Metabolism, Diabetology and Nephrology, Fukushima Medical University Aizu Medical Center
2. Radiation Medical Science Center for the Fukushima Health Management Survey, Fukushima Medical University
[A member of the Comprehensive Health Check and Health Promotion Expert Committee]

**Hata, Kenichi**
1. Meiji Hospital
2. Fukushima Society of Obstetricians and Gynecologists
[None]

**Hayashi, Fumikazu**
1. Office of Epidemiology, Radiation Medical Science Center for the Fukushima Health Management Survey, Fukushima Medical University
2. Department of Epidemiology, Fukushima Medical University School of Medicine
[An observer of the Thyroid Data Analysis Expert Committee, the Comprehensive Health Check and Health Promotion Expert Committee, the Mental Health/Life Style Survey and Care Expert Committee, and the Database System Expert Committee]

**Horikoshi, Naoko**
1. Office of the Mental Health and Lifestyle Survey and Care, Radiation Medical Science Center for the Fukushima Health Management Survey, Fukushima Medical University
2. Department of Public Health, Fukushima Medical University School of Medicine
[A member of the Mental Health/Life Style Survey and Care Expert Committee]

**Hosoya, Mitsuaki**
1. Department of Pediatrics, Fukushima Medical University School of Medicine
2. Office of the Comprehensive Health Check and Health Promotion, Radiation Medical Science Center for the Fukushima Health Management Survey, Fukushima Medical University
[A member of the Basic Survey and Radiation Dose Estimates Expert Committee, the Thyroid Examination Expert Committee, the Comprehensive Health Check and Health Promotion Expert Committee, and the Mental Health/Life Style Survey and Care Expert Committee]

**Ishii, Kayoko**
1. Department of Midwifery and Maternal Nursing, Fukushima Medical University School of Nursing
2. Office of the Pregnancy and Birth Survey, Radiation Medical Science Center for the Fukushima Health Management Survey, Fukushima Medical University
[An observer of the Pregnancy and Birth Survey Expert Committee]

**Ishikawa, Tetsuo**
1. Department of Radiation Physics and Chemistry, Fukushima Medical University School of Medicine
2. Office of the Basic Survey and Radiation Dose Estimates, Radiation Medical Science Center for the Fukushima Health Management Survey, Fukushima Medical University
3. Office of the Information Management and Statistics, Radiation Medical Science Center for the Fukushima Health Management Survey, Fukushima Medical University
[A member of the Basic Survey and Radiation Dose Estimates Expert Committee and the Thyroid Examination Expert Committee]

**Itagaki, Shuntaro**
1. Department of Neuropsychiatry, Fukushima Medical University School of Medicine
2. Fukushima Medical University Health Center, Fukushima Medical University
3. Radiation Medical Science Center for the Fukushima Health Management Survey, Fukushima Medical University
[A member of the Mental Health/Life Style Survey and Care Expert Committee]

**Iwadate, Manabu**
1. Minamisouma Municipal General Hospital
2. Department of Thyroid and Endocrinology, Fukushima Medical University School of Medicine
[An observer of the Thyroid Data Analysis Expert Committee]

**Kamiya, Kenji**
1. Radiation Medical Science Center for the Fukushima Health Management Survey, Fukushima Medical University
2. Research Institute for Radiation Biology and Medicine, Hiroshima University
[A member of the Basic Survey and Radiation Dose Estimates Expert Committee]

**Kawasaki, Yukihiko**
1. Department of Pediatrics, Fukushima General Rehabilitation Center
2. Radiation Medical Science Center for the Fukushima Health Management Survey, Fukushima Medical University
[An observer of the Comprehensive Health Check and Health Promotion Expert Committee]

**Kazama, J. Junichiro**
1. Department of Nephrology and Hypertension, Fukushima Medical University School of Medicine
2. Office of the Comprehensive Health Check and Health Promotion, Radiation Medical Science Center for the Fukushima Health Management Survey, Fukushima Medical University
[A member of the Comprehensive Health Check and Health Promotion Expert Committee; An observer of the Mental Health/Life Style Survey and Care Expert Committee]

**Kobashi, Gen**
1. Department of Public Health, Dokkyo Medical University School of Medicine
2. Radiation Medical Science Center for the Fukushima Health Management Survey, Fukushima Medical University
[A member of the Comprehensive Health Check and Health Promotion Expert Committee; An observer of the Basic Survey and Radiation Dose Estimates Expert Committee]

**Kurihara, Osamu**
1. National Institutes for Quantum Science and Technology
[A member of the Basic Survey and Radiation Dose Estimates Expert Committee]

**Kyozuka, Hyo**
1. Ohta Nishinouchi Hospital
2. Department of Obstetrics and Gynecology, Fukushima Medical University School of Medicine
[An observer of the Pregnancy and Birth Survey Expert Committee]

**Maeda, Masaharu**
1. Department of Disaster Psychiatry, Fukushima Medical University School of Medicine
2. Office of the Mental Health and Lifestyle Survey and Care, Radiation Medical Science Center for the Fukushima Health Management Survey, Fukushima Medical University
[A member of the Mental Health/Life Style Survey and Care Expert Committee, the Pregnancy and Birth Survey Expert Committee, the Risk Communication Evaluation Expert Committee, and the Database System Expert Committee]

**Matsuzuka, Takashi**
1. Office of the Thyroid Ultrasound Examinations, Radiation Medical Science Center for the Fukushima Health Management Survey, Fukushima Medical University
[A member of the Thyroid Data Analysis Expert Committee]

**Miura, Itaru**
1. Department of Neuropsychiatry, Fukushima Medical University School of Medicine
2. Radiation Medical Science Center for the Fukushima Health Management Survey, Fukushima Medical University
[A member of the Mental Health/Life Style Survey and Care Expert Committee]

**Mizuki, Rie**
1. Office of the Mental Health and Lifestyle Survey and Care, Radiation Medical Science Center for the Fukushima Health Management Survey, Fukushima Medical University
[An observer of the Mental Health/Life Style Survey and Care Expert Committee]

**Momoi, Maho**
1. Office of the Mental Health and Lifestyle Survey and Care, Radiation Medical Science Center for the Fukushima Health Management Survey, Fukushima Medical University
2. Department of Disaster Psychiatry, Fukushima Medical University School of Medicine
[A member of the Mental Health/Life Style Survey and Care Expert Committee; An observer of the Comprehensive Health Check and Health Promotion Expert Committee]

**Murata, Tsuyoshi**
1. Shirakawa Kosei General Hospital
2. Department of Obstetrics and Gynecology, Fukushima Medical University School of Medicine
[An observer of the Pregnancy and Birth Survey Expert Committee]

**Nagao, Masanori**
1. Office of Epidemiology, Radiation Medical Science Center for the Fukushima Health Management Survey, Fukushima Medical University
2. Department of Epidemiology, Fukushima Medical University School of Medicine
[An observer of the Thyroid Data Analysis Expert Committee, the Comprehensive Health Check and Health Promotion Expert Committee, and the Mental Health/Life Style Survey and Care Expert Committee]

**Nakai, Akihito**
1. Nippon Medical School Tamanagayama Hospital
2. Radiation Medical Science Center for the Fukushima Health Management Survey, Fukushima Medical University
[A member of the Pregnancy and Birth Survey Expert Committee]

**Nakano, Hironori**
1. Office of the Information Management and Statistics, Radiation Medical Science Center for the Fukushima Health Management Survey, Fukushima Medical University
2. Office of Epidemiology, Radiation Medical Science Center for the Fukushima Health Management Survey, Fukushima Medical University
3. Department of Epidemiology, Fukushima Medical University School of Medicine
[An observer of the Basic Survey and Radiation Dose Estimates Expert Committee, the Comprehensive Health Check and Health Promotion Expert Committee, the Mental Health/Life Style Survey and Care Expert Committee, and the Pregnancy and Birth Survey Expert Committee]

**Nakaya, Tomoki**
1. Graduate School of Environmental Studies, Tohoku University
2. Office of Epidemiology, Radiation Medical Science Center for the Fukushima Health Management Survey, Fukushima Medical University
[An observer of the Database System Expert Committee]

**Ohira, Tetsuya**
1. Department of Epidemiology, Fukushima Medical University School of Medicine
2. Office of Epidemiology, Radiation Medical Science Center for the Fukushima Health Management Survey, Fukushima Medical University
[A member of the Basic Survey and Radiation Dose Estimates Expert Committee, the Thyroid Examination Expert Committee, the Thyroid Data Analysis Expert Committee, the Comprehensive Health Check and Health Promotion Expert Committee, the Mental Health/Life Style Survey and Care Expert Committee, the Pregnancy and Birth Survey Expert Committee, the Risk Communication Evaluation Expert Committee, the Database System Expert Committee, and the Cancer Registry Expert Committee]

**Ohto, Hitoshi**
1. Radiation Medical Science Center for the Fukushima Health Management Survey, Fukushima Medical University
2. Department of Blood Transfusion and Transplantation Immunology, Fukushima Medical University School of Medicine
[A member of the Basic Survey and Radiation Dose Estimates Expert Committee, and the Comprehensive Health Check and Health Promotion Expert Committee; An observer of the Risk Communication Evaluation Expert Committee]

**Oikawa, Yuichi**
1. Office of the Mental Health and Lifestyle Survey and Care, Radiation Medical Science Center for the Fukushima Health Management Survey, Fukushima Medical University
[An observer of the Mental Health/Life Style Survey and Care Expert Committee]

**Okazaki, Kanako**
1. Department of Physical Therapy, Fukushima Medical University School of Health Sciences
2. Office of Epidemiology, Radiation Medical Science Center for the Fukushima Health Management Survey, Fukushima Medical University
[An observer of the Comprehensive Health Check and Health Promotion Expert Committee, the Mental Health/Life Style Survey and Care Expert Committee, and the Pregnancy and Birth Survey Expert Committee]

**Ota, Misao**
1. Preparatory Office for Establishment of Midwifery Training Program, Fukushima Medical University
2. Office of the Pregnancy and Birth Survey, Radiation Medical Science Center for the Fukushima Health Management Survey, Fukushima Medical University
[A member of the Pregnancy and Birth Survey Expert Committee]

**Sakai, Akira**
1. Department of Radiation Life Sciences, Fukushima Medical University School of Medicine
2. Office of the Basic Survey and Radiation Dose Estimates, Radiation Medical Science Center for the Fukushima Health Management Survey, Fukushima Medical University
3. Office of the Comprehensive Health Check and Health Promotion, Radiation Medical Science Center for the Fukushima Health Management Survey, Fukushima Medical University
[A member of the Basic Survey and Radiation Dose Estimates Expert Committee, the Thyroid Data Analysis Expert Committee, and the Comprehensive Health Check and Health Promotion Expert Committee; An observer of the Mental Health/Life Style Survey and Care Expert Committee]

**Sakata, Ritsu**
1. Department of Epidemiology, Radiation Effects Research Foundation
[None]

**Sato, Hideki**
1. Department of Disaster Psychiatry, Fukushima Medical University School of Medicine
2. Office of the Mental Health and Lifestyle Survey and Care, Radiation Medical Science Center for the Fukushima Health Management Survey, Fukushima Medical University
[An observer of the Mental Health/Life Style Survey and Care Expert Committee]

**Satoh, Hiroaki**
1. Department of Diabetes and Metabolism, Juntendo University Urayasu Hospital
2. Radiation Medical Science Center for the Fukushima Health Management Survey, Fukushima Medical University
[A member of the Comprehensive Health Check and Health Promotion Expert Committee]

**Setou, Noriko**
1. Department of Disaster Psychiatry, Fukushima Medical University School of Medicine
2. Office of the Thyroid Ultrasound Examinations, Radiation Medical Science Center for the Fukushima Health Management Survey, Fukushima Medical University
3. Office of the Mental Health and Lifestyle Survey and Care, Radiation Medical Science Center for the Fukushima Health Management Survey, Fukushima Medical University
[A member of the Thyroid Examination Expert Committee; An observer of the Mental Health/Life Style Survey and Care Expert Committee]

**Shimabukuro, Michio**
1. Department of Diabetes, Endocrinology and Metabolism, Fukushima Medical University School of Medicine
2. Office of the Comprehensive Health Check and Health Promotion, Radiation Medical Science Center for the Fukushima Health Management Survey, Fukushima Medical University
[A member of the Thyroid Data Analysis Expert Committee, the Comprehensive Health Check and Health Promotion Expert Committee, the Mental Health/Life Style Survey and Care Expert Committee, the Risk Communication Evaluation Expert Committee, and the Database System Expert Committee]

**Shimura, Hiroki**
1. Department of Laboratory Medicine, Fukushima Medical University School of Medicine
2. Office of the Thyroid Ultrasound Examinations, Radiation Medical Science Center for the Fukushima Health Management Survey, Fukushima Medical University
[A member of the Thyroid Examination Expert Committee, the Thyroid Data Analysis Expert Committee, and the Risk Communication Evaluation Expert Committee]

**Suzuki, Kohta**
1. Department of Health and Psychosocial Medicine, Aichi Medical University School of Medicine
2. Radiation Medical Science Center for the Fukushima Health Management Survey, Fukushima Medical University
[A member of the Pregnancy and Birth Survey Expert Committee]

**Suzuki, Satoru**
1. Office of the Thyroid Ultrasound Examinations, Radiation Medical Science Center for the Fukushima Health Management Survey, Fukushima Medical University
[A member of the Thyroid Examination Expert Committee, the Thyroid Data Analysis Expert Committee, and the Database System Expert Committee]

**Suzuki, Satoshi**
1. Office of the Thyroid Ultrasound Examinations, Radiation Medical Science Center for the Fukushima Health Management Survey, Fukushima Medical University
[A member of the Thyroid Examination Expert Committee and the Thyroid Data Analysis Expert Committee]

**Suzuki, Shinichi**
1. Department of Thyroid Treatment, Fukushima Medical University School of Medicine
[A member of the Thyroid Data Analysis Expert Committee]

**Takahashi, Atsushi**
1. Department of Gastroenterology, Fukushima Medical University School of Medicine
2. Office of the Comprehensive Health Check and Health Promotion, Radiation Medical Science Center for the Fukushima Health Management Survey, Fukushima Medical University
3. Office of the Mental Health and Lifestyle Survey and Care, Radiation Medical Science Center for the Fukushima Health Management Survey, Fukushima Medical University
[A member of the Comprehensive Health Check and Health Promotion Expert Committee and the Mental Health/Life Style Survey and Care Expert Committee]

**Takahashi, Hideto**
1. National Institute of Public Health
2. Radiation Medical Science Center for the Fukushima Health Management Survey, Fukushima Medical University
[A member of the Thyroid Data Analysis Expert Committee and the Comprehensive Health Check and Health Promotion Expert Committee; An observer of the Mental Health/Life Style Survey and Care Expert Committee and the Database System Expert Committee]

**Takahashi, Kunihiko**
1. Department of Biostatistics, M&D Data Science Center, Tokyo Medical and Dental University
2. Office of Epidemiology, Radiation Medical Science Center for the Fukushima Health Management Survey, Fukushima Medical University
[An observer of the Thyroid Data Analysis Expert Committee and the Database System Expert Committee]

**Takebayashi, Yui**
1. Department of Disaster Psychiatry, Fukushima Medical University School of Medicine
2. Office of the Mental Health and Lifestyle Survey and Care, Radiation Medical Science Center for the Fukushima Health Management Survey, Fukushima Medical University
[An observer of the Mental Health/Life Style Survey and Care Expert Committee]

**Yabe, Hirooki**
1. Department of Neuropsychiatry, Fukushima Medical University School of Medicine
2. Radiation Medical Science Center for the Fukushima Health Management Survey, Fukushima Medical University
[A member of the Mental Health/Life Style Survey and Care Expert Committee]

**Yasuda, Shun**
1. Department of Perinatology and Pediatrics for Regional Medical Support, Fukushima Medical University School of Medicine
2. Office of the Pregnancy and Birth Survey, Radiation Medical Science Center for the Fukushima Health Management Survey, Fukushima Medical University
[A member of the Pregnancy and Birth Survey Expert Committee]

**Yasumura, Seiji**
1. Department of Public Health, Fukushima Medical University School of Medicine
2. Radiation Medical Science Center for the Fukushima Health Management Survey, Fukushima Medical University
[An observer of the Basic Survey and Radiation Dose Estimates Expert Committee, the Thyroid Data Analysis Expert Committee, the Comprehensive Health Check and Health Promotion Expert Committee, the Mental Health/Life Style Survey and Care Expert Committee, the Pregnancy and Birth Survey Expert Committee, the Risk Communication Evaluation Expert Committee, the Database System Expert Committee, and the Cancer Registry Expert Committee]

**Yokoya, Susumu**
1. Thyroid and Endocrine Center, Fukushima Medical University
2. Office of the Thyroid Ultrasound Examinations, Radiation Medical Science Center for the Fukushima Health Management Survey, Fukushima Medical University
[A member of the Thyroid Examination Expert Committee and the Thyroid Data Analysis Expert Committee]

**Yonai, Shunsuke**
1. National Institutes for Quantum Science and Technology
[None]

**Yoshida-Komiya, Hiromi**
1. Center for Gender-Specific Medicine, Fukushima Medical University
[An observer of the Pregnancy and Birth Survey Expert Committee]

